# Bee Venom Components as Therapeutic Tools against Prostate Cancer

**DOI:** 10.3390/toxins13050337

**Published:** 2021-05-07

**Authors:** Jasmin Katrin Badawi

**Affiliations:** Medical Faculty Mannheim of the Ruprecht-Karls-University of Heidelberg, Theodor-Kutzer-Ufer 1–3, D-68167 Mannheim, Germany; jkb.ch@bluewin.ch; Tel.: +0041-77-47-16-981

**Keywords:** bee venom, prostate cancer, melittin

## Abstract

Prostate cancer is one of the most common cancers in men. Despite the development of a variety of therapeutic agents to treat either metastatic hormone-sensitive prostate cancer, advanced prostate cancer, or nonmetastatic/metastatic castration-resistant prostate cancer, the progression or spread of the disease often cannot be avoided. Additionally, the development of resistance of prostate cancer cells to available therapeutic agents is a well-known problem. Despite extensive and cost-intensive research over decades, curative therapy for metastatic prostate cancer is still not available. Therefore, additional therapeutic agents are still needed. The animal kingdom offers a valuable source of natural substances used for the treatment of a variety of diseases. Bee venom of the honeybee is a mixture of many components. It contains proteins acting as enzymes such as phospholipase A2, smaller proteins and peptides such as melittin and apamin, phospholipids, and physiologically active amines such as histamine, dopamine, and noradrenaline. Melittin has been shown to induce apoptosis in different cancer cell lines, including prostate cancer cell lines. It also influences cell proliferation, angiogenesis, and necrosis as well as motility, migration, metastasis, and invasion of tumour cells. Hence, it represents an interesting anticancer agent. In this review article, studies about the effect of bee venom components on prostate cancer cells are discussed. An electronic literature research was performed utilising PubMed in February 2021. All scientific publications, which examine this interesting subject, are discussed. Furthermore, the different types of application of these promising substances are outlined. The studies clearly indicate that bee venom or melittin exhibited anticancer effects in various prostate cancer cell lines and in xenografts. In most of the studies, a combination of bee venom or the modified melittin with another molecule was utilised in order to avoid side effects and, additionally, to target selectively the prostate cancer cells or the surrounding tissue. The studies showed that systemic side effects and unwanted damage to healthy tissue and organs could be minimised when the anticancer drug was not activated until binding to the cancer cells or the surrounding tissue. Different targets were used, such as the matrix metalloproteinase 2, hormone receptors expressed by prostate cancer cells, the extracellular domain of PSMA, and the fibroblast activation protein occurring in the stroma of prostate cancer cells. Another approach used loaded phosphate micelles, which were cleaved by the enzyme secretory phospholipase A2 produced by prostate cancer cells. In a totally different approach, targeted nanoparticles containing the melittin gene were used for prostate cancer gene therapy. By the targeted nonviral gene delivery, the gene encoding melittin was delivered to the prostate cancer cells without systemic side effects. This review of the scientific literature reveals totally different approaches using bee venom, melittin, modified melittin, or protoxin as anticancer agents. The toxic agents acted through several different mechanisms to produce their anti-prostate cancer effects. These mechanisms are not fully understood yet and more experimental studies are necessary to reveal the complete mode of action. Nevertheless, the researchers have conducted pioneering work. Based on these results, further experimental and clinical studies about melittin and modifications of this interesting agent deriving from nature are necessary and could possibly lead to a complementary treatment option for prostate cancer.

## 1. Introduction

Prostate cancer is one of the most frequent cancers in men. Even in metastatic hormone-sensitive prostate cancer (mHSPC), the traditionally used monotherapy with androgens (androgen deprivation therapy: ADT) was changed to a multidrug approach. Some of these drugs used in addition to ADT were originally developed for the treatment of castration-resistant or metastatic prostate cancer. Taxanes such as docetaxel and cabazitaxel [1], the androgen biosynthesis inhibitor abiraterone acetate [2], a selective inhibitor of the 17α-hydroxylase and C17,20-lyase enzymatic activities of cytochrome P450 (CYP) 17, and the androgen receptor pathway inhibitors enzalutamide [3], darolutamide, and apalutamide [4] are all agents used for the treatment of prostate cancer.

Prostate cancer is a very heterogeneous disease. More and more subtypes are currently being identified, and therefore, which prostate cancer therapy will be used depends on the subtype as well.

A currently introduced additional pharmacological class are the Poly (ADP-ribose) polymerase (PARP) inhibitors, to which belongs olaparib [5]. The enzyme PARP is important in deoxyribonucleic acid (DNA) repair, which includes the repair of DNA damage inside cancer cells after chemotherapy. Targeting and inhibiting this enzyme could increase the sensitivity to chemotherapy and may lead to the cell death of cancer cells. Olaparib delays the cancer progression but only for several months.

Radium-223 [6] or other radionuclides [7] can be used in addition when bone metastases occur. Another new therapeutical approach is the use of radioligand therapy in prostate cancer, such as 177Lu-PSMA-617 [8].

Furthermore, checkpoint inhibitors such as pembrolizumab are examined in clinical trials for selected cases [9].

Nevertheless, more and bigger studies concerning these recently developed drugs are needed, and only subgroups of patients will benefit from these various new therapeutic approaches. The progression or spread of the disease often cannot be avoided despite the above-described development of a variety of additional therapeutic agents to treat metastatic hormone-sensitive prostate cancer (mHSPC), advanced prostate cancer, and nonmetastatic/metastatic castration-resistant prostate cancer (CRPC). Additionally, the resistance of prostate cancer cells to available therapeutic agents is a well-known problem, for example, resistance to taxane chemotherapy [10] or enzalutamide resistance [11].

Despite extensive and cost-intensive research over decades, curative therapy for metastatic prostate cancer is still not available. Judging by the variety of ongoing clinical studies which indicate how new drugs are currently searched for, additional therapeutic agents are still needed and should be warmly welcomed.

The plant kingdom provides useful drugs for the treatment of cancer in human beings such as the worldwide-used pharmacological class of taxanes, originally deriving from the bark of a yew tree (botanical nomenclature: Taxus). Additionally, bacteria from the animal kingdom are valuable sources of substances, which can be used for the treatment of a variety of diseases. For example, the well-known bacterial toxin botulinum toxin is used to treat the hyperactive urinary bladder in adults and children [12].

Toxins derived from animals could also be valuable therapeutic tools to treat urological cancers.

Bee venom (BV) of the honeybee is a mixture of many components. It contains proteins acting as enzymes such as phospholipase A2, a strong allergen with a high molecular weight, phospholipase B, acid phosphomonoesterase, hyaluronidase, phosphatase, lysophospholipase, and smaller proteins and peptides such as melittin, apamin, adolapin, tertiapin, and secapin. Furthermore, bee venom contains phospholipids and physiologically active amines such as histamine, dopamine, and noradrenaline [13]. Further components are amino acids, sugars such as glucose and fructose, pheromones and minerals such as calcium and magnesium [13].

The major peptide component of bee venom is melittin (40–50% of the dry venom), which is composed of 26 amino acids [13]. It is membrane active, diminishes surface tension of membranes, stimulates smooth muscles, increases the capillary permeability, lowers blood coagulation, and acts as an immunostimulatory or immunosuppressive agent [13]. Higher doses are inflammatory and haemolytic [13]. Besides the antiparasitic, antibacterial, antiviral, and anti-inflammatory effects of melittin, the anticancer activities are of special interest for clinical applications [14,15]. Melittin was shown to induce apoptosis in different human leukaemia cell lines [16] and in other cancer cell lines. Furthermore, it influences cell proliferation, angiogenesis, and necrosis, in addition to motility, migration, metastasis, and invasion of tumour cells [17]. The mechanisms of action of melittin are complex and not fully understood. Melittin was shown to affect signal transduction and different regulatory pathways. For instance, melittin inhibited the JAK2/STAT3 pathway, induced NF-κB inactivation, and influenced the matrix metalloproteinase pathway and caspases-mediated pathways [17]. Since these mechanisms of action complement each other, melittin represents a remarkably interesting anticancer agent.

Jo et al. [18] showed the anticancer effects of bee venom toxin and melittin in ovarian cancer cells through induction of death receptors and inhibition of the JAK2/STAT3 pathway (JAK2/STAT3: Janus Kinase 2/signal transducer and activator of transcription 3). In breast cancer cells, melittin was shown to suppress the EGF-induced cell motility (EGF: epidermal growth factor) and invasion by inhibiting the PI3K/Akt/mTOR (PI3K: phosphatidylinositol-3-kinase, Akt: protein kinase B, mTOR: mammalian target of rapamycin) signalling pathway [19].

Yang et al. [20] showed that melittin inhibited the proliferation of malignant human glioma cells and induced apoptosis. Li et al. [21] proved that melittin induced a growth arrest and apoptosis of the human hepatocellular carcinoma cell line BEL-7402. Liu et al. [22] showed that melittin prevented liver cancer cell metastasis through inhibition of the Rac1-dependent pathway. Honeybee venom induced a calcium-dependent apoptotic cell death in human melanoma A2058 cells [23]. Furthermore, bee venom was shown to inhibit colon cancer cell growth by activation of death receptors and inhibition of nuclear factor kappa [24].

In this review, published in vitro and in vivo studies about the treatment of prostate cancer by bee venom components are discussed. This substance class is not very well known at this point, and publications about the effect of bee venom components on prostate cancer cells are rare.

## 2. Results

Using the terms “bee venom” and “prostate cancer”, 22 articles were found. Using the terms “carcinoma” instead of “cancer” did not lead to additional publications. Using the terms “melittin” and “prostate cancer”, 19 articles were found.

After exclusion using the above-mentioned search criteria, 17 articles concerning prostate cancer were found. In the study of Gross and Andrä [25], melittin was only used as a reference peptide. The focus of this study was on investigating the preferred interaction sites of other antimicrobial peptides with PC-3 prostate cancer cells. Therefore, this study is not included in the detail of the discussion section.

Finally, 16 articles were included in the discussion section inclusive of 2 review articles. A summary of these articles is given in Table 1.

Furthermore, additional publications are cited for a better understanding of the scientific context.

## 3. Discussion

Park et al. [26] performed different in vitro and in vivo experiments on prostate cancer cells. The results were promising. Bee venom and melittin were used in different concentrations. They inhibited the cell growth concentration- and time-dependently in the following prostate cancer cell lines: LNCaP, DU145, and PC-3 prostate cancer cells. The inhibitory effect was bigger in PC-3 and DU145 cells than in LNCaP cells.

In another experiment, the induction of apoptotic cell death by treatment with bee venom and melittin in different concentrations was proven, respectively. Both modalities of treatment led to a decrease in the expression of the following antiapoptotic proteins: Bcl-2, X-chromosome linked inhibitor of apoptosis protein (XIAP), cellular inhibitor of apoptosis protein 2 (cIAP2), inducible nitric oxide synthase (iNOS), cyclooxygenase-2 (COX-2), and cytosolic phospholipase A2 (cPLA2). The expression of proapoptotic proteins, such as the cleaved forms of caspase-3 and -9, was increased by bee venom or melittin, respectively. Involvement of the NF-kB signalling pathway in the apoptotic cell death by bee venom and melittin was also shown. The authors concluded that that the caspase pathway is involved in the bee venom and melittin-induced apoptotic cell death through inactivation of NF-kB.

Additionally, in vivo studies were performed in male nude mice. To induce tumour growth, PC-3 prostate cancer cells were injected subcutaneously into the right lower flanks of the mice. After the tumours had reached a defined average volume, bee venom in a concentration of 3 and 6 mg/kg was injected intraperitoneally twice per week for 4 weeks. In a positive control group, cisplatin at a concentration of 5 mg/kg was injected once a week. A sham group was used as the control group. The tumour growth gradually and time-dependently decreased during the treatment with bee venom. The volumes of the tumours were significantly smaller in both bee-venom-treated groups, compared to the sham control group. Additionally, in the group treated with cisplatin the volumes of the tumours were smaller. The number of apoptotic cells was dose-dependently increased in the groups treated with bee venom in two different concentrations. The bee venom inhibited the DNA binding activity of NF-kB in vivo and the translocation of p50 and p65 in tumour tissue. Drug resistance in cancer cells is a general problem, also occurring in prostate cancer cells. Several chemotherapeutic agents were shown to induce the activation of NF-kB in cancer cells. This mechanism is suspected to be partly responsible for the resistance to the drug [27]. Therefore, bee venom components may be useful in the treatment of prostate cancer that is resistant to chemotherapy. Additionally, bee venom used at the higher concentration did not induce serious health problems in the animals.

In following different studies, bee venom or its main component melittin was not used alone but rather in combination with another molecule to target prostate cancer cells and to reduce side effects.

### 3.1. Targeting Matrix Metalloproteinase 2

This approach was chosen by Holle et al. [28], who performed in vitro and in vivo experiments. The authors coupled the biotinylated melittin peptide with avidin in order to receive an inactive peptide, which was first activated after binding to cancer cells.

Cancer cells were shown [29,30] to express matrix metalloproteinase 2 (MMP2). Therefore, the MMP2 target sequence was incorporated into the peptide. The goal was to target selected cells expressing MMP2. The authors performed experiments in vitro and in vivo. These in vitro experiments on DU 145 prostate cancer cell lines showed that the melittin–avidin-conjugate induced cell lysis of the used prostate cancer cell line. Notably, the conjugate did not lead to significant cell lysis of normal mouse fibroblast cells (L-cells). Furthermore, it was shown that the MMP2 activity of the prostate cancer cell line DU 145 was significantly higher in comparison to L-cells (sevenfold higher). In addition to the in vivo experiments, the melittin–avidin conjugate was examined in vivo in mice inoculated with melanoma cells. Briefly, 14 days after the subcutaneous inoculation, 6 mice were treated with the melittin–avidin conjugate, which was injected into the tumour daily for 2 weeks, and 6 mice were not treated and were used as the control group. On day 25, the conjugate-treated tumours were significantly smaller than the tumours of the control group. Even though only a small group of animals were examined, and the duration of the treatment was short, the results were promising. The cytotoxic effect of the melittin-containing conjugate on cancer cells with higher activity of MMP2 was proven in this study, for example, in the investigated prostate cancer cell line and the additionally investigated ovarian cancer cell line (SK-OV-3 cells). These results point to the possible use of melittin as a pharmacological tool against prostate cancer.

### 3.2. Targeting Special Hormone Receptors of Prostate Cancer Cells

Luteinising hormone (LH) and human chorionic gonadotropin (hCG) belong to the glycoprotein hormone family. They consist of noncovalently associated α- and β-subunits [31]. Prostate cancer cells are known to express specific LH/CG (CG: chorionic gonadotropin) and luteinising hormone-releasing hormone (LHRH) receptors [32].

Therefore, conjugates enclosing structures of CG or LHRH target these cells and may be useful for prostate cancer therapy.

Hansel et al. [33] performed different in vivo and in vitro experiments using conjugates of membrane disrupting lytic peptides and a segment of the beta-chain of CG or LHRH. The lytic peptides linked to these hormones were shown to destroy prostate cancer cells. One of the lytic peptides used was a synthetic analogue of melittin called hecate.

In 2001, Leuschner et al. [34] prepared a conjugate of hecate and a 15-amino acid segment of the beta-chain of LH and added it in vitro to cultures of prostate cancer cells. Concentration-dependent toxicity was shown for different prostate cancer cell lines according to the capacity of the LH receptor. The following rank order was found: BRF41T > DU145 > PC-3 > LNCaP. The experiments were performed in the presence and absence of steroids and other admixtures. When steroids were removed, the sensitivity to the drug was reduced in most of the cell lines (BRF41T, PC-3, LNCaP) up to 50% except for the DU 145 cells. This result suggests that steroids regulate the LH expression in prostate cancer cells. Additionally, PC-3 xenografts of male athymic nude mice were used for in vivo experiments. The conjugate of hecate and the segment of beta-LH at a dose of 12 mg/kg was applied once a week for 3 weeks via the lateral tail vein. A reduction of the tumour burden was found. The histological examination revealed necrotic cells and fluid inside the tumours of the treated animals, whereas the tumours of the untreated control group consisted of sheets of cells with large hyperchromatic nuclei and prominent nucleoli. Inside the tumours of the untreated animals, many mitotic figures were found, and richness of blood vessels was proven. In contrast, the tumours of the treated animals were poorly vascularised. The other organs (such as liver, heart, lung kidney, pituitary) showed no histological abnormalities except for the testes: The interstitial cells were shrunken, and primary and secondary spermatocytes were nearly absent from the tubules. These changes resemble the changes induced by gonadotropin-releasing hormone (GnRH) agonists used to reduce the secretion of testosterone in patients with prostate cancer.

Further similar experiments were performed by Leuschner et al. [35], using a conjugate of the lytic peptide hecate and LHRH based on the knowledge that human prostate xenografts also express LHRH receptors.

The authors examined the ability of this conjugate to destroy PC-3 cells in vitro and in vivo. For that purpose, the conjugate of hecate and LHRH was added to different cultures of prostate cancer cells (PC-3, BRF 41 T, DU145, LNCaP cells). In some of the experiments, steroids were present, while in others, no steroids were added. The authors found that the sensitivity of LNCaP and PC-3 cells to the conjugate was decreased when steroids were removed from the culture media, whereas the addition of oestrogen restored the sensitivity. For the in vivo experiments, nude male mice were treated with the conjugate after the establishment of PC-3 xenografts. The injections of LHRH-hecate led to an arrest of the tumour growth. Furthermore, a significant reduction of the tumour burden from 62.2 mg/g body weight in the control group versus 10.5 mg/g body weight in the treated mice was found. The treatment with unconjugated LHRH and hecate showed no effect on the tumour burden and the tumour viability, suggesting that hecate was responsible for this effect. In the treated group, necrosis of the tumour was found.

The conjugation of lytic peptide to a fragment of beta-LH or to LHRH may be an effective treatment for prostate cancer, but further studies are necessary.

Similar results were published by Zaleska et al. [36]. The authors also used a conjugate of hecate and a 15-amino acid segment of the beta-chain of chorionic gonadotropin (CG) to perform in vitro experiments in different cell lines. Three cell lines consisting of physiologically existing cells and two cell lines consisting of tumour cells were examined in vitro. In both groups, LH/CG receptor-positive and LH/CG receptor-negative cell lines were regarded concerning the cytotoxic effect of the conjugate described above at different concentrations. In the three cell lines being receptor-positive, the number of LH/CG receptors was determined, too. The two cell lines not possessing LH/CG receptors were used as controls. The toxicity of the conjugate was concentration-dependent in all cell types. Furthermore, the conjugate was more cytotoxic in the cell lines possessing LH/CG receptors. Another result of these first series of experiments was that the toxicity of the conjugate was dependent on the number of binding sites for LH/CG which suggests that the conjugate selectively kills cells expressing these receptors. As receptor-positive cancer cell line, the Leydig cancer cell line BLT-1 was used.

In the second series of experiments [37], a human prostate cancer cell line (PC-3) was used for in vitro studies. The presence of LH receptors in PC-3 cells was confirmed in this study. The cytotoxic effect was determined by measuring the release of lactate dehydrogenase. The conjugate caused a significantly higher release of this enzyme compared with hecate alone at all effective doses (1, 5, and 10 µM). The lysis of the prostate cancer cell membranes induced by the conjugate occurred after 15 min of incubation leading to uncontrolled leaks of cellular components. At the low concentration of 1 µM, the conjugate induced an ionic imbalance in the cells. The effect of preincubation of the PC-3 cells with CG on LDH activity was also examined. The toxicity of the conjugate examined at all concentrations was decreased after preincubation of the PC-3 cells with CG used at a dose of 100 ng/mL. Used at a lower dose of 10 ng/mL, CG was not effective.

This result could be interpreted as competition between the hecate-beta–CG conjugate and CG for the LH/CG receptors.

Additional experiments were performed to examine if hecate and the conjugate induce apoptosis of the cancer cells. The apoptotic DNA fragmentation is considered a key feature of apoptosis. Consequently, a DNA laddering assay was performed by using a 1.5% agarose gel electrophoresis. It was shown that hecate alone and the conjugate used at different concentrations did not induce fragmentation of DNA. Since apoptotic DNA fragmentation is considered a key feature of apoptosis, the induced cell deaths of the prostate cancer cells by hecate and the conjugate were more likely based on necrosis.

Other researchers investigated the effect of melittin on other cell lines, such as leukaemia cells, and showed that this natural substance induced apoptosis [14]. There may be a difference concerning the lytic mechanism between the natural compound of bee venom melittin containing 26 amino acids and hecate, which is a 23-amino acid synthetic peptide analogue of melittin.

In animals in which benign prostatic hyperplasia was induced, the application of bee venom was shown to decrease the levels of the antiapoptotic proteins and increase the levels of proapoptotic factors [38].

Bogacki et al. [39] investigated if the peptides hecate-betaCG and Phor14-betaCG(ala) induced the immune system of mice to produce antibodies, which would be disadvantageous for anticancer drugs. No specific antibodies were produced in the treated animals after the injection of the substances, indicating that these peptides were not immunogenic. Furthermore, it was shown that both peptides led to degenerative changes in the prostate glands and testes of the treated male animals. This finding is in accordance with the histopathological results of other studies.

### 3.3. Using Immunoconjugates, in Which Monoclonal Antibodies Targeting Prostate Cancer Cells and a Modified Melittin Molecule Were Conjugated

Russell et al. [40] investigated the cytotoxic properties of immunoconjugates against prostate cancer cells in vitro and in vivo. For creating the conjugates, the authors used a toxin deriving from melittin and different monoclonal antibodies targeting prostate cancer cells. The authors modified the structure of native melittin because it contains a highly cationic C-terminus with potential immunogenic and allergenic properties. The modified molecule called peptide 101 had a truncated C-terminus without this region. Using this way, its water solubility was enhanced in comparison to melittin. Peptide 101 was cross-linked to a special monoclonal antibody of the mouse, J591 or BLCA-38, known for targeting prostate cancer cells. J591 belongs to the immunoglobulin G (IgG) class and recognises the extracellular domain of prostate-specific membrane antigen (PSMA), which is a cell surface peptidase highly expressed by malignant prostate epithelial cells [41] and vascular endothelial cells of numerous solid tumour malignancies. It was shown in clinical trials that radiolabelled J591 accurately targets bone and soft tissue metastatic prostate cancer sites [42].

The second monoclonal antibody used to target prostate cancer cells was BLCA-38. It was shown to bind to many human prostate cancer cells but not to normal cells [40]. Male nude mice bearing subcutaneous human prostate cancer xenografts (LNCaP-LN3 or DU-145, respectively) were used for the in vivo experiments. The authors showed that the systemic or intratumoural injection of immunoconjugates inhibited the growth of the tumour and led to improved survival. The conjugates induced necrosis and haemorrhage in the tumours. However, new growth of tumour cells around necrotic areas occurred in some cases. No toxicity was found in other organs examined histologically, such as liver, kidney, or heart. Interestingly, the application of the toxin without being conjugated to an antibody was less effective. The dose was limited due to the solubility of the conjugate.

In clinical practice, such conjugates may only be useful to target tumours of low burden, for instance, after decreasing the volume of the tumour with other methods or to target smaller metastases. Further modifications of the structure of the conjugates may also be necessary to increase the solubility of the conjugate so that higher dosages could be used in vivo.

Carter et al. [43] investigated the antibody BLCA-38 in comparison to the antibody J591 in vivo. The antibodies were radio-iodinated using I125 (iodine-125) and applied systemically by intraperitoneal administration in athymic nude mice in which subcutaneous prostate cancer xenografts were induced. The xenografts of the mice treated with radio-iodinated BLCA-38 consisted of human DU-145 prostate cancer cells. The xenografts of the mice treated with radio-iodinated J591consisted of human LNCaP-LN3 prostate cancer cells. The results of these experiments suggested that radio-iodinated BLCA-38 showed a comparable localisation within DU-145 xenografts to that of J591 within LNCaP-LN3 xenografts. Additionally, in this field of research, further studies are necessary.

### 3.4. Role of the Bee Venom Secretory Phospholipase A2 as Tool against Prostate Cancer

Putz et al. [44] investigated if tumour cell growth of the prostate cancer cell line DU145 and other cancer cell lines was inhibited by the secretory phospholipase A2 deriving from bee venom (bv-sPLA2) and phosphatidylinositol-(3,4)-bisphosphate. Both substances were investigated separately and in combination. A combination of these substances lysed the tumours. The lysates generated by this method were shown to enhance the maturation and immunostimulatory capacity of monocyte-derived dendritic cells. Further studies are necessary to prove if these substances could be useful tools for the immunotherapy of prostate cancer and other malignant tumours.

### 3.5. Phosphate Micelles as Possible Delivery System for Prostate Cancer Cells

The development of effective delivery systems for anticancer drugs facilitates their utilisation in vivo. Sharipov et al. [45] developed special phosphate micelles and loaded them with upconversion nanoparticles (UCPN). By performing in vitro studies on different cell lines, it was shown that the UCNPs were selectively released to prostate cancer cells. The loaded phosphate micelles were cleaved by the secretory phospholipase A2 enzyme. This enzyme catalyses the hydrolysis of phospholipids. The expression of the secretory phospholipase A2 was shown to increase with the progression of the cancer cells to androgen independence [46]. In the study of Sharipov et al. [45], the micelles were used to deliver selectively UCPN to 22Rv1 prostate cancer cell lines.

These micelles could be used for imaging prostate cancer tissue. Whether anticancer drugs instead of imaging agents could be transported to cancer cells using loaded micelles remains to be established. Further investigation should also be focused on the compatibility of such micelles inside the human body.

### 3.6. Interaction of Melittin and S100P in Prostate Cancer Cells

Gribenko et al. [47] investigated the conformational and thermodynamic properties of binding of peptides to the human S100P protein using the peptide melittin.

The authors showed that S100P and melittin interact in a Ca2+-dependent and -independent manner. S100 represents a multigenic family of nonubiquitous Ca2+-modulated proteins of the EF-hand type expressed in vertebrates [48]. EF-hand is a diverse motif class consisting of amino acids folding into a helix–loop–helix structure.

Since S100P is associated with prostate cancer progression [49], the interaction of melittin and S100P may be of special interest for the anticancer effect of melittin against prostate cancer.

### 3.7. Targeting the Fibroblast Activation Protein of the Stroma of Prostate Cancer Tissue by a Promelittin Protoxin

Another interesting experimental approach is to target the prostate cancer stroma with a fibroblast activation protein-activated (FAP-activated) promelittin protoxin [50]. For this purpose, the authors generated a modified promelittin peptide, in which FAP substrate sequences were introduced into the prodomain. FAP is a cell–surface serine protease [51] detected in the stroma of different human epithelial cancers [52], including the stroma of prostate cancer [53]. In the honeybee, melittin is secreted into the venom glands as promelittin. During the activation process, a stepwise cleavage of the pro part of promelittin by the enzyme dipeptidyl peptidase 4 (DPP4) occurs [54]. LeBeau et al. [50] changed the structure of the promelittin peptide and developed a modified structure of promelittin, in which the prodomain is removable by a non-DPP4-like endopeptidase such as FAP. In this way, the toxic melittin is generated in tissues, in which FAP is present, leading to the death of both stromal cells inside the tumour and tumour cells and endothelial cells surrounding the stromal cells. For the in vivo studies, the modified promelittin was injected in LNCaP human prostate xenografts in host animals (male nude mice). The maximum tolerated dose of intratumoural melittin was shown to be 5.7 mg/kg. The intratumoural dose of 40 mg/kg of the modified promelittin was well tolerated, and a dose of 200 mg/kg was lethal in about 33% of the treated animals. Two different groups of animals were treated with the above-described doses. They received a single intratumoural injection and the tumours were imaged over 34 days. The tumours developed a necrotic centre and overlying eschar. Complete regressions only occurred in selected animals of the group treated with the higher dose of the modified promelittin. The authors showed that a single dose of 40 mg/kg of the modified promelittin was well tolerated when applied intratumorally, but that the same dose was lethal when it was applied intravenously.

For clinical use, the systemic application would be preferable. Only in selected cases, direct injection into the tumour may be useful. If a modified protoxin could be developed, which does not lead to haemolysis when applied systemically, it could be used as a therapeutic tool. Other enzymes than FAP, which are prostate cancer specific, may be used to activate a modified protoxin of melittin, as well. Further investigations of these interesting issues are needed.

### 3.8. Targeted Nonviral Gene Delivery by Using the Melittin Gene for Prostate Cancer Gene Therapy

In order to avoid systemic side effects of a protoxin or the toxin, the targeted nonviral gene delivery could be used alternatively. This method is an interesting approach to treat cancer by gene therapy [55]. In this way, exogenous genes can be transferred to malignant human prostate cells [56], for example, to cause cell deaths. Besides an appropriate vector to transfer the genes [57], which has a high transfection efficiency, an appropriate cancer cell targeting ligand [58] is necessary to deliver the gene encoding an appropriate cytotoxic peptide to the cancer cells. Bee venom components could be an appropriate cytotoxic peptide.

In an interesting study by Tarokh et al. [59], the melittin gene was used for prostate cancer gene therapy. The authors developed a chlorotoxin (CTX)-targeted nanovector for the delivery of the gene encoding melittin, which is known to inhibit the growth of prostate cancer cells. The peptide CTX consisting of 36 amino acids was originally isolated from scorpion venom [60].

It binds specifically to the MMP-2, which is a part of a complex, and inhibits the enzymatic activity of it. In cancer cells, which overexpress MMP-2, CTX was shown to be a promising tumour-targeting ligand [61]. Tarokh et al. [59] investigated the cytotoxic effects of CTX-targeted nanoparticles containing the melittin gene on two different cell lines: the PC3 human prostate cancer cell line being metalloproteinase-2 positive (ATCC CRL-1435) and the NIH3T3 fibroblast cell line (ATCC CRL-1658) being metalloproteinase-2 negative. Furthermore, targeted and nontargeted nanoparticles were compared. The transfection efficiency of targeted nanoparticles was significantly higher than that of non-targeted nanoparticles. The targeted nanoparticles containing the melittin gene showed high cytotoxicity on PC3 cells but no toxicity on the NIH3T3 cell line. These results suggest that targeted nanoparticles containing the melittin gene could be promising agents for prostate cancer gene therapy.

## 4. Conclusions

The existing studies clearly indicate that bee venom or melittin exhibited anticancer effects in various prostate cancer cell lines and in xenografts.

Although Park et al. [23] showed that in vivo experiments in mice had no serious health problems after the injection of bee venom, in most of the studies a combination of bee venom or the modified melittin with another molecule was used in order to avoid side effects and to target selectively the prostate cancer cells or the surrounding tissue. If the anticancer drug is not activated until binding to the cancer cells or the surrounding tissue, systemic side effects can be minimised, and unwanted damage to healthy tissue and organs can be avoided.

Targeting the matrix metalloproteinase 2 expressed by cancer cells was shown to be effective in vitro and in a mouse model [25].

In several studies, hormone receptors expressed by prostate cancer cells were used as targets. For this purpose, a synthetic analogue of melittin was linked to the physiological ligand, for example, to LHRH or to a segment of the beta-chain of CG. In vitro and in vivo experiments [31,32,33,34] in a mouse model showed anticancer activity without affecting most of the organs except for the testes [31]. In mice [36], no specific antibodies against the peptides were produced. Therefore, such conjugates are promising tools for the treatment of prostate cancer subtypes, which express the correspondent hormone receptors. Further in vivo studies in appropriate models and eventually clinical studies in patients with prostate cancer are necessary to draw conclusions about the clinical usefulness of these conjugates.

Another pharmacological approach is the use of modified melittin conjugated to monoclonal antibodies targeting prostate cancer cells. By modifying the structure of melittin, the potentially immunogenic and allergenic properties of melittin could be reduced and the water solubility should be improved [37]. Two different antibodies were used: J591, which recognises the extracellular domain of PSMA, and the antibody BLCA-38. The systemic and intratumoural injection inhibited the growth of the tumour and led to an improved survival without being toxic to other organs. Since the solubility of the conjugate limits the dosage, these conjugates may only be useful to target tumours of low burden or smaller metastases. More experimental studies about modifications of the structure of conjugates being more hydrophilic could lead to the development of drugs, which are also usable for bigger tumour burdens.

It was shown in vitro that special phosphate micelles loaded with UCPN were selectively released to prostate cancer cells [42]. The loaded phosphate micelles were cleaved by an enzyme of the prostate cancer cells, the secretory phospholipase A2. Since the expression of the group IIa secretory phospholipase A2 increases, when cancer cells progress to androgen independency, such a delivery system, could be used to treat a progressive type of prostate cancer. If anticancer drugs could be transported to the cancer cells instead of imaging agents, such micelles would represent an effective delivery system. Further research should also be focused on the compatibility and safety of such micelles for the human body.

In another interesting experimental approach, the fibroblast activation protein of the stroma of prostate cancer cells was used as a target. A modified promelittin peptide was injected in an animal model. The toxic melittin is generated in tissues, in which FAP is present. FAP acts as an activating enzyme. Since the intratumoural application was better tolerated than the systemic application, this method is not usable in metastatic disease. If a modified protoxin could be developed, which does not lead to haemolysis when applied systemically, it could be used as a therapeutic tool. Other enzymes than FAP, which are prostate cancer specific, may also be used to activate a modified protoxin of melittin. Further investigations of these interesting issues are needed.

In order to avoid systemic side effects of a protoxin or the toxin, the targeted non-viral gene delivery represents an excellent method to deliver the gene encoding melittin to the prostate cancer cells. Chlorotoxin was used as the tumour-targeting ligand. This peptide was originally isolated from scorpion venom. It binds specifically to the matrix matalloproteinase-2 (MMP-2), which is part of a complex. The targeted nanoparticles containing the melittin gene showed high cytotoxicity on PC3 cells but no toxicity on the NIH3T3 cell line. These results suggest that targeted nanoparticles containing the melittin gene could be promising agents for prostate cancer gene therapy.

The review of the literature reveals totally different approaches using bee venom, melittin, modified melittin, or a protoxin as anticancer agents. The toxic agents acted through several different mechanisms to produce their anti-prostate cancer effects. These mechanisms are not fully understood yet, and more experimental studies are necessary to reveal the complete mode of action.

Nevertheless, the researchers have pioneered significant new possibilities.

Based on these results, further experimental and clinical studies about melittin and modifications of this interesting agent should be conducted to substantiate the applicability of these agents for prostate cancer treatment further. In this way, a complementary treatment option for prostate cancer can be developed using modified toxins originally derived from nature.

## 5. Material and Methods

In February 2021, an electronic literature search was performed using PubMed. Following search terms were combined: “bee venom” and “prostate cancer”. Instead of the term “cancer”, the term “carcinoma” was also used in the above-mentioned combinations. Additionally, the following combination was used: “melittin” and “prostate cancer”. First, the identified titles and abstracts were screened. Then, the full text was screened for exclusion. The following studies were excluded:

1. Articles in which the subject was non-oncological;
2. Articles in which the subject was a non-urological tumour, for example, leukaemia, or in which cancers other than prostate cancers were examined.
3. Review articles and consensus reports.

In this review, all types of studies about the effect of bee venom components on prostate cancer cells are included since the number of studies on the subject is rare and important information about the subject would be ignored by focusing only on randomised (prospective) studies.

## Figures and Tables

**Table 1:** A summary of the original articles about the effects of bee venom, melittin, and derivatives on prostate cancer cells is provided here.

First Author	Year of Publication	Type of Experiment	Anti-Cancer Agent	Effects	Mechanism	Side Effects/Dosage	Other Characteristics/Targets
Park et al.	2011	in vitro: LNCaP, DU145, and PC-3 cells in vivo: male nude mice with PC-3 xenografts; treatment with bee venom intraperitoneally for 4 weeks	bee venom, melittin	in vitro: inhibition of the cell growth concentration- and time-dependently in vivo: tumour growth gradually and time-dependently decreased; number of apoptotic cells increased dose-dependently	decrease of the expression of the following antiapoptotic proteins: Bcl-2, XIAP (X-chromosome linked inhibitor of apoptosis protein), cIAP2 (cellular inhibitor of apoptosis protein 2), iNOS (inducible nitric oxide synthase), COX-2 (cyclooxygenase-2), and cPLA2 (cytosolic phospholipase A2) - increase of the expression of pro-apoptotic proteins (cleaved forms of caspase-3 and -9) - involvement of the NF-kB signalling pathway in the apoptotic cell death - inhibition of the DNA binding activity of NF-kB (nuclear factor kappa-light-chain-enhancer of activated B cells) in vivo and of the translocation of p50 and p65 in tumour tissue	Bee venom used at the higher concentration did not induce serious health problems in the animals.	
Holle et al.	2002	in vitro experiments on DU 145 prostate cancer cell lines, in vivo experiments in mice inoculated with melanoma cells; conjugate injected into the tumour for 2 weeks	biotinylated melittin peptide coupled with avidin; MMP2 target sequence was incorporated into the peptide	in vitro: melittin-avidin-conjugate induced cell lysis of the prostate cancer cell line; no significant cell lysis of normal mouse fibroblast cells (L-cells) in vivo: at day 25 the conjugate-treated tumours were significantly smaller			cytotoxic effect of the conjugate on cancer cells with higher activity of MMP2 was proven in the investigated prostate cancer cell line and the additionally investigated ovarian cancer cell line (SK-OV-3 cells).
Leuschner et al.	2001	in vitro in vivo: PC-3 xenografts in male athymic nude, systemic treatment for 3 weeks	conjugate of hecate and a 15-amino acid segment of the beta-chain of LH (luteinising hormone)	in vitro: concentration-dependent toxicity for different prostate cancer cell lines according to the capacity of the LH receptor; rank order: BRF41T > DU145 > PC-3 > LNCaP in vivo: reduction of the tumour burden; histological examination: necrotic cells and fluid inside the tumours of the treated animals; tumours of the treated animals: poorly vascularised		In other organs (such as liver, heart, lung kidney, pituitary): no histological abnormalities except for the testes: interstitial cells shrunken, primary and secondary spermatocytes nearly absent from the tubules	When steroids were removed, the sensitivity to the drug was reduced in most of the cell lines (BRF41T, PC-3, LNCaP) up to 50% except for the DU 145 cells.
Leuschner et al.	2003	in vitro: different cultures of prostate cancer cells (PC-3, BRF 41 T, DU145, LNCaP cells) in vivo: in nude male mice: establishment of PC-3 xenografts	conjugate of the lytic peptide hecate and LHRH	in vitro: sensitivity of LNCaP and PC-3 cells to the conjugate decreased, when steroids were removed from the culture media; the addition of oestrogen restored sensitivity. in vivo: arrest of the tumour growth¸ significant reduction of the tumour burden; necrosis of the tumour			
Zaleska et al.	2003	in vitro experiments in different cell lines	conjugate of hecate and a 15-amino acid segment of the beta-chain of chorionic gonadotropin	toxicity of the conjugate concentration-dependent in all cell types: conjugate was more cytotoxic in cell lines possessing LH/CG receptors; toxicity of the conjugate was dependent on the number of binding sites for LH/CG			
Bodek et al.	2005	in vitro: human prostate cancer cell line (PC-3)	hecate-CG-beta conjugate	conjugate caused significantly higher release of LDH compared with hecate alone at all effective doses (1, 5, and 10 μM); lysis of prostate cancer cell membranes occurred after 15 min of incubation; hecate alone and the conjugate at different concentrations did not induce fragmentation of DNA.	induction of necrosis more likely		
Bogacki et al.	2008	in vivo: in mice: investigation about the induction of antibodies by hecate-betaCG	hecate-betaCG	no specific antibodies produced in treated animals after injection. degenerative changes in the prostate glands and testes			
Russell et al.	2004	in vivo: male nude mice bearing subcutaneous human prostate cancer xenografts (LNCaP-LN3 or DU-145)	conjugates of a modification of native melittin called Peptide 101 and different monoclonal antibodies targeting prostate cancer cells	systemic or intra-tumoral injection of immunoconjugates inhibited growth of the tumour, improved survival; induction of necrosis and haemorrhage in the tumours; new growth of tumour cells around necrotic areas occurred in some cases; application of the toxin without conjugation to an antibody: less effective		Histological examination: no toxicity found in other organs such as liver, kidney, or the heart	Used antibodies: J591 or BLCA-38; J591 recognises the extracellular domain of prostate-specific membrane antigen (PSMA); dose limitation due to solubility of the conjugate
Carter et al.	2004	in vivo: intraperitoneal administration in athymic nude mice in which subcutaneous prostate cancer xenografts were induced (human DU-145 or human LNCaP-LN3 prostate cancer cells)	Investigation of the antibody BLCA-38 in comparison to the antibody J591	radio-iodinated BLCA-38 showed a comparable localisation within DU-145 xenografts to that of J591 within LNCaP-LN3 xenografts			No involvement of bee venom or melittin in this study.
Putz et al.	2006	in vitro	bee venom secretory phospholipase A2 (bv-sPLA2); additionally, phosphatidylinositol-(3,4)-bisphosphate was examined	Bv-sPLA2 or phosphatidylinositol-(3,4)-bisphosphate alone: moderate effects on the proliferation of A498 renal cell carcinoma cells, T-47D breast cancer cells, DU145 prostate cancer cells, and BEAS-2B transformed lung cells; bv-sPLA2 was co-administered with phosphatidylinositol-(3,4)-bisphosphate: potent inhibition of [3H] thymidine incorporation into all tested cell lines			tumour cell lysates generated with bv-sPLA2 and phosphatidylinositol-(3,4)-bisphosphate induced maturation of human moDCs
Sharipov et al.	2017	in vitro	Investigation of a special delivery system. Special micelles were used to deliver selectively UCPN (upconversion nanoparticles) to 22Rv1 prostate cancer cell lines.	UCPNs were selectively released to prostate cancer cells. Loaded phosphate micelles were cleaved by the secretory phospholipase A2 enzyme.			No direct involvement of bee venom or melittin, but transport system may be useful to deliver anti-cancer drugs.
Gribenko et al.	2002	in vitro	Investigation of the conformational and thermodynamic properties of binding of peptides to the human S100P protein using the peptide melittin	S100P and melittin interact in a Ca2+-dependent and -independent manner.			Since S100P is associated with prostate cancer progression, the interaction of melittin and S100P may be of special interest for the anti-cancer effect of melittin against prostate cancer.
LeBeau et al.	2009	in vivo: LNCaP human prostate xenografts in male nude mice; single intratumoural injection, tumours were imaged over 34 days	fibroblast activation protein-activated (FAP-activated) promelittin protoxin	Complete regressions: only in selected animals treated with the higher dose; single dose of 40 mg/kg: well tolerated, when applied intratumourally; same dose: lethal, when applied intravenously		maximum tolerated dose of intratumoural melittin: 5.7 mg/kg; intratumoural dose of 40 mg/kg: well-tolerated; dose of 200 mg/kg: lethal in about 33% of the treated animals	Target: the fibroblast activation protein of the stroma of prostate cancer tissue
Tarokh et al.	2017	in vitro: 2 different cell lines: PC3 human prostate cancer cells (metalloproteinase-2 positive) and the NIH3T3 fibroblast cell line (metalloproteinase-2 negative)	chlorotoxin (CTX)-targeted nanovector for the delivery of the gene encoding melittin	transfection efficiency of targeted nanoparticles: significantly higher than that of non-targeted nanoparticles; targeted nanoparticles containing the melittin gene: high cytotoxicity on PC3 cells, no toxicity on the NIH3T3 cell line			prostate cancer gene therapy; binding specifically to the matrix matalloproteinase-2 (MMP-2)
